# Diffuse Optical Tomography Activation in the Somatosensory Cortex: Specific Activation by Painful vs. Non-Painful Thermal Stimuli

**DOI:** 10.1371/journal.pone.0008016

**Published:** 2009-11-24

**Authors:** Lino Becerra, Will Harris, Margaret Grant, Edward George, David Boas, David Borsook

**Affiliations:** 1 Pain and Analgesia Imaging Neuroscience (P.A.I.N.) Group, McLean Hospital, Belmont, Massachusetts, United States of America; 2 Photon Migration Laboratory, McLean Hospital, Belmont, Massachusetts, United States of America; 3 McLean Hospital, Belmont, Massachusetts, United States of America; 4 Massachusetts General Hospital, Charlestown, Massachusetts, United States of America; 5 Athinoula A. Martinos Center for Biomedical Engineering, Harvard Medical School, Charlestown, Massachusetts, United States of America; University of Sydney, Australia

## Abstract

**Background:**

Pain is difficult to assess due to the subjective nature of self-reporting. The lack of objective measures of pain has hampered the development of new treatments as well as the evaluation of current ones. Functional MRI studies of pain have begun to delineate potential brain response signatures that could be used as objective read-outs of pain. Using Diffuse Optical Tomography (DOT), we have shown in the past a distinct DOT signal over the somatosensory cortex to a noxious heat stimulus that could be distinguished from the signal elicited by innocuous mechanical stimuli. Here we further our findings by studying the response to thermal innocuous and noxious stimuli.

**Methodology/Principal Findings:**

Innocuous and noxious thermal stimuli were applied to the skin of the face of the first division (ophthalmic) of the trigeminal nerve in healthy volunteers (N = 6). Stimuli temperatures were adjusted for each subject to evoke warm (equivalent to a 3/10) and painful hot (7/10) sensations in a verbal rating scale (0/10 = no/max pain). A set of 26 stimuli (5 sec each) was applied for each temperature with inter-stimulus intervals varied between 8 and 15 sec using a Peltier thermode. A DOT system was used to capture cortical responses on both sides of the head over the primary somatosensory cortical region (S1). For the innocuous stimuli, group results indicated mainly activation on the contralateral side with a weak ipsilateral response. For the noxious stimuli, bilateral activation was observed with comparable amplitudes on both sides. Furthermore, noxious stimuli produced a temporal biphasic response while innocuous stimuli produced a monophasic response.

**Conclusions/Significance:**

These results are in accordance with fMRI and our other DOT studies of innocuous mechanical and noxious heat stimuli. The data indicate the differentiation of DOT cortical responses for pain vs. innocuous stimuli that may be useful in assessing objectively acute pain.

## Introduction

Recent work in the field of neuroimaging (fMRI) of pain has suggested some potentially specific biomarkers for pain and analgesics. By using a non-invasive easily applied system (DOT) to measure cortical brain activity in a similar manner to fMRI we can evaluate specific brain responses in chronic pain conditions, in which evoked pain reflects the patient's symptoms, and make an assessment of pain intensity levels. Furthermore, this approach can be used to study the response to analgesics in an outpatient setting. At this time, measures of pain or response to analgesics are dependent on self-reports. “*Amid the difficulties and uncertainties of investigating drug action in man and attempting to quantify drug effect, visual analogue scales, without the need for complex equipment and difficult experiments, have emerged as a tempting prospect*” [1]. The development of objective measures will allow for a quantifiable measure of pain and analgesia. Currently no objective measure exists.

Previously we have used DOT to evaluate painful thermal (heat) and non-painful mechanical stimulation (brush) in healthy volunteers [2]. Following stimulation to the dorsum of the hand, we detected biphasic activation in the somatosensory cortex to noxious heat stimuli and monophasic activation to tactile (brush) stimulation. In addition, fMRI studies of similar stimuli [3], [4] also demonstrated a single response for brush and a biphasic one for noxious heat. However, the previous report had a confounding component that restricted our ability to clearly state the origin of the biphasic response; both stimulus nature (thermal vs. mechanical) and perception (noxious vs. innocuous) were changed.

In this report, we wish the define experimentally the nature of the biphasic response by removing one of the confounding variables of our previous experiments, namely, limiting the stimuli nature to thermal and comparing noxious vs. innocuous perceptions.

## Methods

### Subjects

Six healthy volunteers were recruited through local advertisements. All were right-handed males of 18–40 years in age. Subjects with a history of neurological trauma, neurological or psychiatric disorders, or diabetes were excluded. Subjects were also excluded if they were taking any psychoactive medications or were unable to keep their head still for a period of 360 consecutive seconds. Written informed consent was obtained from all subjects according to the guidelines established by the Massachusetts General Hospital Institutional Review Board who reviewed and approved this study. Subjects were compensated for their participation.

### Equipment

The equipment has been described in detail elsewhere [5]. Briefly, a multichannel continuous wave optical imager (CW5, TechEn Inc., Milford, MA) was used to emit the two wavelengths of light, 690 nm and 830 nm. These two wavelengths are used to measure changes in cortical deoxyhemoglobin (HbR) and oxyhemoglobin (HbO) concentration via differential absorption characteristics of the two wavelengths of light by these two molecules. The head probe used in this study consisted of 26 sources and 26 detectors (see Becerra et al., 2008). Source fibers emitting the 690 nm wavelength were paired off with those emitting the 830 nm wavelength to form an “optode.” The main probe was arranged with one central, anterior-posterior row of 6 optodes per hemisphere. Each row of optodes was flanked on either side by a row of 6 detectors strategically placed 3 cm away from the sources in order to measure activation at cortical depth. Additionally, 2 optodes were placed on the forehead in order obtain prefrontal cortex activation. These two source optodes were similarly flanked on either side by single detectors.

Subjects remained sitting in a reclined position for the duration of the experiment. Lights were turned off in the room during data acquisition to minimize signal contamination from ambient light sources.

### Thermal Stimuli Thresholds

A Peltier-based computer controlled thermal probe (3×3 cm) was used for these experiments (TSA-II, Medoc Haifa, Israel). For each subject, the probe was attached to the face and the temperature of the probe was ramped up at 1.5C/s until the subject declared that the probe had achieved a warm non-painful temperature. They were instructed that in a scale of 0–10 (VAS), that level corresponded to a 3. After repeating the procedure 3 times, the temperatures were averaged and the average was used in the experiments. For the noxious stimuli, the probe's temperature was ramped in a similar fashion but subjects were instructed to stop the ramp once the pain intensity reached a level of 7/10. The procedure was repeated 3 times and the average temperature was used in the experiments.

### Paradigm

The thermal probe was set at the correct temperature for the experiment (VAS of 3 or 7) and applied to the face of the subject upon prompting and removed at the end of each stimulus. Care was taken to apply the probe to the same site with the same pressure. The paradigm consisted of 26 stimuli of 5 second duration over 6 minutes with a jittered inter-stimulus interval (ISI) 0f 6–13 seconds and average ISI of 8.5 seconds. The paradigm was applied twice for each stimulus type. Prompts to apply stimuli were presented audibly via headphones to the investigator but not to the subject.

### Data Analysis

Analysis was carried out using the open source software Homer [6], that is implemented in Matlab (Mathworks, Natick, MA). The analysis has been described in detail elsewhere [5]. Briefly, optical data were demodulated to identify source-detector pair signals; signal intensities were normalized to provide a relative change of intensity. Data were then low-pass filtered to eliminate cardiac pulsatile effects. The change in optical density was calculated for each wavelength, and finally, the changes in oxy- and deoxyhemoglobin were calculated using the modified Beer-Lambert Law [6]. Source-detector pairs were inspected for gross- signal changes induced by movement; signal changes larger than 20 µM were eliminated. Single trial averages (STA's) were calculated for each source-detector pair for the oxy- (HbO) and deoxyhemoglobin (Hb) concentration changes. This was achieved by deconvoluting the paradigm from the responses and temporally aligning responses for each stimulus delivered before averaging (see Becerra et al. 2008). All the figures in this article display changes in oxyhemoglobin (in µM) from baseline (no stimulation).

**Figure 1:**
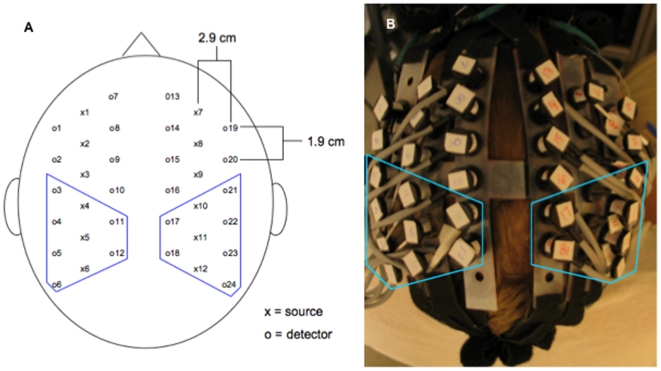
(A) Schematic of source-detector arrangement over a subject's head and (B) corresponding photograph. Source-detector pairs inside the polygons were inspected and data were extracted from those displaying a localized response.

The resulting data were displayed spatially for each source-detector pair, and the signal corresponding to the somatosensory cortex was identified as adjacent areas of activation around the detectors receiving light from source 4 plus or minus 1 source on the contralateral hemisphere to the stimulated side (see **Figure 1A and 1B**). For simplicity, this activation is referred to in the manuscript as S1 activation. The signal corresponding to ipsilateral S1 was identified as the mirroring ipsilateral source-detector pairings corresponding to those considered to be S1 on the contralateral side.

### Statistical Analysis

**Figure 2:**
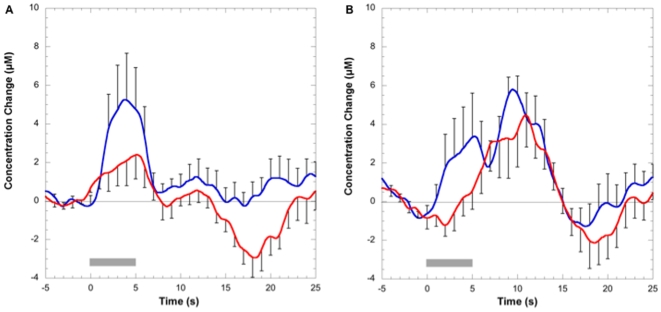
(A) Left Panel: Group average response to innocuous thermal stimuli in the contralateral (blue line) and ipsilateral side (red line). (B) Right Panel: Group average response to noxious stimuli for contralateral and ipsilateral sides. Both graphs display changes in oxyhemoglobin concentration. Error bars represent the SEM. Gray block indicates the duration of the applied stimuli.

**Figure 3:**
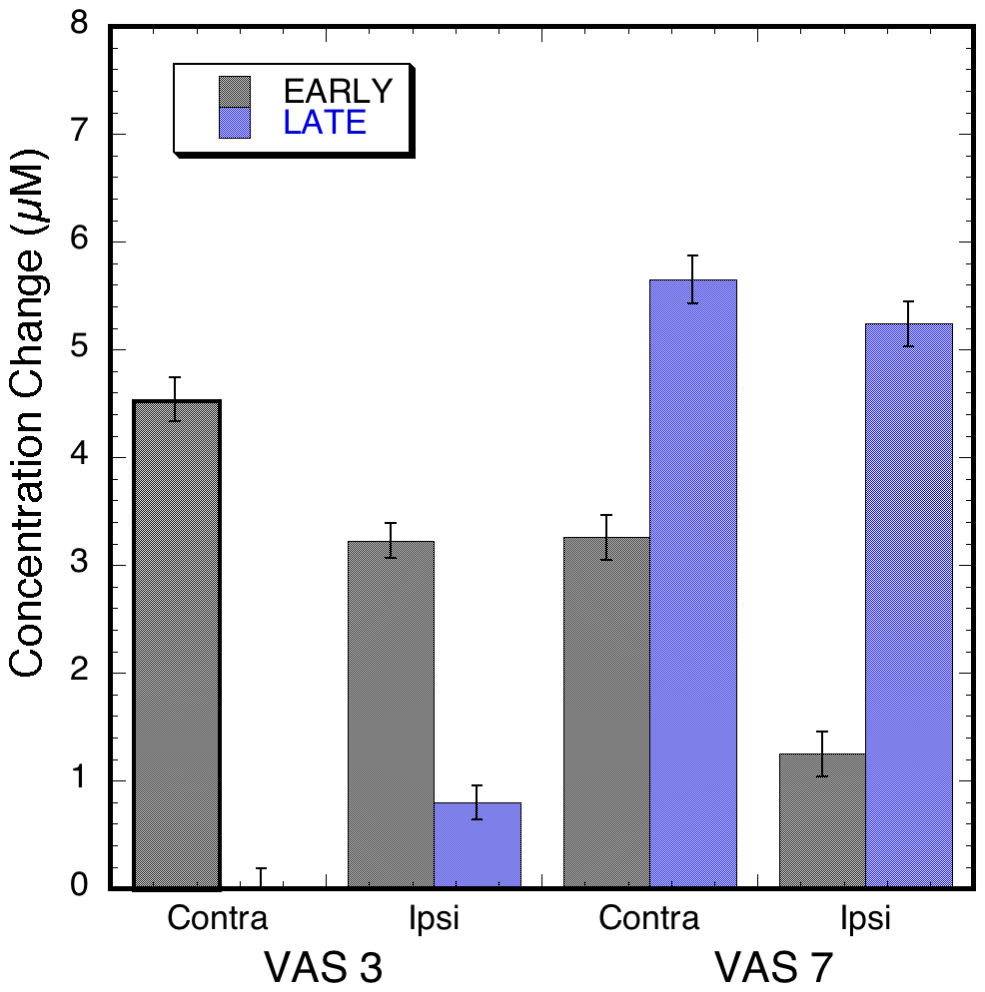
Average amplitude of the Early and Late phase in the ipsilateral and contralateral sensorimotor area in response to innocuous (VAS = 3) and noxious (VAS = 7) stimuli. Error bars represent the SEM.

For each experiment, identified S1 individual STAs were averaged and are shown in Figure 2. A 2-gamma function was used to non-linearly fit (Matlab) the averages. Fitted parameters were used to determine time-to-peak for each experiment and phase (Table 1). The fit values for the noxious heat response on the contralateral side were used to generate two model phases: early and late as previously described. Early and late phases were used as explanatory variables in a generalized linear model fit of each individual response in S1 to assess each phase contribution to the observed response; these values were used to calculate average amplitudes of early and late phases in both experiments (Figure 3). T-tests were used to determine statistically significant difference (or the lack thereof) between early and late phase amplitudes.

**Table 1:** Time-to-peak determined from a 2 gamma function fit of the average responses contra/ipsi for VAS of 3 and 7 (see text).

	VAS-3 contra	VAS-3 ipsi	VAS-7 contra	VAS 7 ipsi
EARLY	3.72	3.23	4.34	7.93
LATE	–	12.06	10.12	11.68

## Results

### Subjects

Subjects included in the study were 26.5±1.55 years of age. No significant artifacts were observed in their data and none were discarded.

### Matching Temperatures and Subjective Pain Ratings

Thermal stimulation was applied in order to obtain subjective ratings of 3/10 (non-painful) and 7/10 (moderate pain) for each participant. The average temperatures used for the 3/10 and 7/10 were 41.4±0.76 (mean±SEM) and 45.3±0.66, respectively (mean±SEM).

### Innocuous Heat Stimulation

The response (Figure 2A) displays a monophasic response, similar to the mechanical innocuous stimulation using a brush. The ipsilateral side also presents a mono-phasic response but smaller compared to the contralateral side, as previously observed for mechanical stimulation. It seems to have a small late component as determined below.

### Noxious Heat Stimulation

Our results indicate (**Figure 2B**) that noxious heat to the face produced a biphasic response as for stimulation of the hand but with a smaller early phase, as previously detected when applied to the hand [2]. The ipsilateral response is similar in size to the contralateral one as observed before. Nonlinear fits to a 2-gamma function resulted in time-to-peak values similar to the ones observed before [2] and displayed in **Table 1**.

### Early and Late Phase Amplitudes for Innocuous and Noxious Heat

Results from the GLM analysis of phases' amplitudes are depicted in **Figure 3** **.** For the innocuous results the contralateral early phase amplitude was significant larger that the ipsilateral one (p<0.0005). The early phase amplitudes (contra- and ipsilateral) were larger than the late phase amplitudes, respectively (p<0.0001). For the noxious heat results, the contralateral early phase amplitude was significantly larger than the ipsilateral amplitude (p< 0.0001), similar to what was observed in the innocuous results. However, the contralateral late phase was not significantly different from the ipsilateral one (p = 0.2).

## Discussion

### Summary of Findings

Innocuous and noxious heat produced a mono- and bi-phasic response, similar to results to innocuous mechanical and noxious thermal stimuli. Time-to-peak values for the early and late phase are similar to those reported previously, although they seem shorter in these experiments, likely due to differences in conduction distances since the previous experiment's stimuli were applied to the hand and here to the face. Innocuous and noxious stimuli produced a more pronounced contralateral activation in the early phase while noxious heat displayed bilateral activation of similar magnitude in the late phase. Quantification of early and late components revealed that the innocuous experiment produced mainly an early phase response. The noxious displayed a late phase larger in size than the early phase. The hemodynamic responses were similar to those obtained when stimuli were applied to the hand.

### Brain Activation Following Painful vs. Non-painful Stimuli

Studies of sensorimotor cortical activation following innocuous stimulation have reported both unilateral [7] as well as bilateral S1 activation [8], [9]. Furthermore, some studies indicate bilateral activation with a prominent component contralateral to the stimulus [10] while others have found inhibition of ipsilateral activation [11]. Taken together, these results seem to indicate that bilateral activation to innocuous stimuli is observed, albeit, sometimes the ipsilateral activation is not reported or varies in terms of its relative strength compared to the contralateral side. Several of these were carried out with electrical stimulation of a main nerve [9]. As a result, multiple fiber subtypes (innocuous and noxious specific subtypes) are stimulated and the observed responses may include nociceptive-related activation.

### S1 Cortex as a Potential Readout for Pain

In this study we detected changes in sensorimotor cortical regions that seem to correspond with the trigeminal representation of the face in the primary sensory cortex. In prior studies using fMRI we have reported specific and somatotopic activation in the S1 region following similar painful thermal stimuli to the three divisions of the face [12], [13] that included the one used here, i.e., the ophthalmic division of the trigeminal nerve also designated as V1 or the first division of the nerve.

Painful stimuli elicited bilateral activation in these studies. It potentially could be attributed to activation in S1 and S2 and recorded together, since both are activated by noxious stimuli [13]. However, the geometry of the optodes setup and the distance from the closest source-detector pair to S2 (more than 3 cm with no source-detector pair across that area of the cortex) would render that contribution to the signal minimal.

A number of methods have been available for measuring pain in clinical practice, clinical trials and research studies. The most common has been the visual analog scale (VAS). However, these ratings may be unreliable because of factors such as study design, expectations and emotional state. The field has been working towards more objective markers including genetic, functional imaging and other formats for pain phenotyping. Here we present one approach that may have applications in evaluating pain because of the specificity of the underlying cortical anatomy (S1 and its subdivisions – see above) and the relative ease of use of DOT to map cortical function. Our results seem to indicate that on the basis of the temporal response in the sensorimotor cortex, it is possible to differentiate thermal noxious from innocuous stimulus (mechanical or thermal). It maybe possible to use that difference to objectively distinguish noxious from innocuous stimulus perception. Having a standardized and more objective measure would allow for a transformational change in measurements of pain in research and in the clinic (e.g., pain reduction from the use of analgesics).

### Conclusions

DOT is finding widespread application in the study of human brain activation, motivating further application-specific development of the technology. Our results seem to indicate that the bi-phasic response is ubiquitous to pain as demonstrated here and in our previous report while a mono-phasic response is characteristic of an innocuous response either to thermal or mechanical stimulation.
